# Dataset of vaccination and confidence in the Malaysian government during Covid-19 pandemic

**DOI:** 10.1016/j.dib.2022.108148

**Published:** 2022-04-11

**Authors:** Chuen Khee Pek, Kah Yung Choy, Kian Kok Toh, Fang Ee Foo, Tat Huei Cham

**Affiliations:** a Tan Sri Omar Centre for Science, Technology and Innovation Policy Studies, Kuala Lumpur, Malaysia; b UCSI Poll Research Centre, Kuala Lumpur, Malaysia; c Faculty of Business and Management, UCSI University, Kuala Lumpur, Malaysia; d UCSI Graduate Business School, UCSI University, Kuala Lumpur, Malaysia

**Keywords:** Covid-19, Vaccination, Government, Economic recovery, Voter's rights

## Abstract

The online poll collected a data set on the confidence sentiments of Malaysians on the following three major areas with their respective events related to the Covid-19 pandemic. Foremost, the survey gathered the data of the respondents on their vaccination registration, and confidence on the efficacy of the available vaccines, respectively. In addition, the respondents were asked of their confidence of an economic recovery with the availability of the vaccines, the government in managing the Covid-19 pandemic, and an economic recovery for the country, respectively. Furthermore, the respondents were asked of their likelihood to exercise their voter's rights before and after herd immunity. The convenience sampling was applied between March and April 2021 and participated by 1,136 respondents. The confidence levels were measured on a Likert-scale of 1 (lowest) to 10 (highest). The mean values were considerably high indicating healthy confidence levels of Malaysians on all the events described. The socio-demographic characteristics of the respondents were also collected. Near 60 percent of Malaysians had registered for the vaccination programme. All the events showed weak significant correlations between them. The correlations between the events and socio-demographic characteristics were analysed and the results showed no correlations between the events and ethnicity, and gender respectively. Although the data of the poll is limited, they can be used as a reference point for researchers, and data review for the future generations to understand the events during the Covid-19 pandemic in Malaysia.

## Specifications Table

**Table:** 

Subject	Social Sciences
Specific subject area	Confidence sentiments of Malaysians on vaccination roll-out, the government and economic recovery, and voter's rights during Covid-19 pandemic.
Type of data	Primary data, Table
How the data were acquired	The poll questionnaire was developed in English. The data were gathered using an online poll platform (Google forms) and was administered by trained pollsters. The questionnaire is attached as a supplementary file.
Data format	Raw, Analyzed
Description of data collection	The poll data was gathered from a convenience sampling of 1,136 respondents with age of 18-year-old and above, living in Malaysia and having access to internet. The poll was conducted using an online poll (Google Forms questionnaires) between March and April 2021.
Data source location	Region: Asia Country: Malaysia
Data accessibility	https://data.mendeley.com/datasets/ymgfx4g9mc/2 doi: 10.17632/ymgfx4g9mc.2

## Value of the Data

• The poll contributes the confidence sentiments of Malaysians in the Covid-19 vaccines and the government's management of the Covid-19 pandemic. Besides, the data also demonstrates the influence of vaccine availability on the economic recovery of Malaysia, and how the people perceived the government's ability in recovering the economy. Furthermore, the data provided insights to the readiness of the people to cast their votes in the coming general elections before and after herd immunity is achieved, respectively.
• The dataset concerns the readiness of Malaysians in accepting the Covid-19 vaccines and serves as a good literature review for the policy makers in the health sectors and future generations. It also records the trust Malaysians have on the available vaccines, the government's ability to manage Covid-19 and economic recovery in the pandemic times.
• The data serves as a reference point for researchers working on a similar topic or area. In the future there may be other pandemics and the findings from this survey record the events during Covid-19 pandemic for comparisons and reference.

## 1 Data Description

The data set was gathered and tested on a convenience sample of 1,136 respondents, who were 18 years old and above, and residents of Malaysia. The respondents answered the questionnaires voluntarily. They were from the 13 states and three federal territories of Malaysia. A poll questionnaire was distributed using an online poll platform between March and April 2021. The questionnaire provided insights of events such as the acceptance of Covid-19 vaccination, levels of confidence in the economic recovery with the availability of the vaccine, the ability of the government in managing Covid-19 and economic recovery, and likelihood of exercising the voter's rights based on herd immunity amongst Malaysians.

The data set comprised of three major areas; the first area asked about the registration status for the vaccination of Covid-19 by the respondents and followed by a 10-point Likert scale system (1: not confident at all to 10: extremely confident) to measure how confident the respondents were with the available vaccines. The second area measured two levels of confidence: economic recovery with the availability of vaccine, and the ability of the government in managing Covid-19, and economic recovery. The third area queries the likelihood of Malaysians in exercising their voter's rights before, and after herd immunity using the same Likert scale system.

The poll showed encouraging response of Malaysians in the registration for vaccination with 57.5 percent indicating they had registered for the vaccination programme, 39 percent stating that they had not and 3.5 percent responding should not vaccinate due to medical reasons.

The socio-demographic characteristics of the respondents are presented in Table 1. The survey questionnaire is provided as a supplementary file.

**Table 1:** Socio-demographic characteristics of respondents (n = 1,136).

Characteristics	n (%)	Mean	Characteristics	n (%)	Mean
Ethnicity		2.03	Area		1.55
Malay	412 (36.3)		Urban	613 (54.0)	
Chinese	418 (36.8)		Semi-urban	426 (37.5)	
Indian	167 (14.7)		Rural	97 (8.50)	
	
Others	139 (12.2)		Generation (Gen)		1.88
	
Gender		1.57	Gen Z (1995-2003)	463 (40.8)	
Male	488 (43.0)		Gen Y (1994-1981)	407 (35.8)	
Female	648 (57.0)		Gen X (1980-1965)	210 (18.5)	
	
State		9.82	Boomers (1964 and older)	56 (4.90)	
	
F.T. Labuan	7 (0.6)		Education level (Edu)		3.70
F.T. Putrajaya	12 (1.1)		No formal education	8 (0.7)	
F.T. Kuala Lumpur	76 (6.7)		Primary school	24 (2.1)	
Johor	125 (11)		Secondary school	266 (23.4)	
Kedah	63 (5.5)		University and equivalent	838 (73.8)	
	
Kelantan	66 (5.8)		Income group (Inc)		1.50
Malacca	50 (4.4)		Bottom 40 (< MYR4,850)	653 (57.5)	
Negeri Sembilan	91 (8.0)		Middle 40 (MYR4,850-10,959)	402 (35.4)	
Pahang	36 (3.2)		Top 20 (> MYR10,959)	81 (7.1)	
Perak	64 (5.6)				
Perlis	24 (2.1)				
Pulau Pinang	56 (4.9)				
Sabah	121 (10.7)				
Sarawak	71 (6.3)				
Selangor	246 (21.7)				
Terengganu	28 (2.5)

**Table 2:** Mean values of events.

Events	n (%)	Mean
VacReg		2.54
Should not vaccinate	40 (3.5)	
No	443 (39.0)	
Yes	653 (57.5)	
ConVac		6.67
ConEcoVac		6.65
ConGovCov		6.50
ConGovEco		6.39
VotB4HI		6.03
VotAftHI		7.08

## 2 Experimental Design, Materials and Methods

Malaysians suffer significant economic and health losses to the Covid-19 pandemic. On 25 January 2020, Malaysia reported the first imported case of Covid-19 from Wuhan, China. The first Malaysian to be tested positive for Covid-19 virus was reported a week after the imported case. Since then, the number of Covid-19 cases have been increasing and created tensed fear amongst Malaysians. There was no specific treatment for Covid-19 and vaccination has been the only hope for the people. In Malaysia, the Pfizer-BionTech vaccines arrived in February 2021 and subsequently the Sinovac vaccines a week thereafter. The first phase of inoculation was for frontline staff starting from 26 February 2021. The second phase was for high-risk groups such as the elderly and those with chronic conditions from April to August 2021, and the third phase was for adults aged 18 and above starting from May 2021 and all the way until February 2022. Before the rollout of the vaccination programme in February 2021, Malaysia recorded the total number of 214,959 Covid-19 cases with 760 deaths on 31 January 2021. As of 20 September 2021, the total number of Covid-19 cases and deaths stood at 2.1 million and 23,443 respectively. The Covid-19 pandemic has brought forth major challenges and caused adversities to the world economy [1]. Malaysia is not spared. The economic growth of Malaysia has been hit badly with the World Bank revising the GDP growth from six percent to 4.5 percent amid a second wave of the Covid-19 virus since mid-April 2021 [2].

The data collected was analysed and discussed hereafter. The number of respondents registered for the vaccination were 653, did not register were 443 and 40 were advised by doctors not to take the vaccination due to medical reasons. The confidence of the Malaysians in the vaccination is important alike the increasing trust in the Black communities to reduce vaccination challenge in the United States in [3]. The mean scores for all the major areas measured were considerably high between 6.03 to 7.08. The public gave a mean score of 6.67 on their confidence with the available vaccines (ConVac) in the country. The confidence of an economic recovery with the availability of vaccine (ConEcoVac), confidence on the government managing Covid-19 pandemic (ConGovVac), and economic recovery (ConGovEco) were measured with means of 6.65, 6.50 and 6.39 respectively, indicating that the people were having a considerably high confidence in the three Events. The people were more likely to cast their votes in the general election after herd immunity (VotAftHI) with mean 7.08 as compared to before herd immunity (VotB4HI) at a mean of 6.03.

Table 3 shows the correlations between the Events indicating weak positive significance in most of the pairs except ConEcoVac-ConGovEco (0.717) and ConGovCov-ConGovEco (0.790). This shows that the confidence in economic recovery with the availability of vaccines (ConEcoVac) and government in recovering the economy (ConGovEco) has a strong positive correlation. The same is with the confidence in the government in managing the Covid-19 pandemic (ConGovCov), and ConGovEco.

**Table 3:** Correlations between events.

Events	VacReg	ConVac	ConEcoVac	ConGovCov	ConGovEco	VotB4HI	VotAftHI
VacReg	1	0.433⁎⁎	0.373⁎⁎	0.294⁎⁎	0.303⁎⁎	0.215⁎⁎	0.284⁎⁎
ConVac	0.433⁎⁎	1	0.672⁎⁎	0.634⁎⁎	0.566⁎⁎	0.376⁎⁎	0.485⁎⁎
ConEcoVac	0.373⁎⁎	0.672⁎⁎	1	0.643⁎⁎	0.717⁎⁎	0.429⁎⁎	0.530⁎⁎
ConGovCov	0.294⁎⁎	0.634⁎⁎	0.643⁎⁎	1	0.790⁎⁎	0.450⁎⁎	0.469⁎⁎
ConGovEco	0.303⁎⁎	0.566⁎⁎	0.717⁎⁎	0.790⁎⁎	1	0.426⁎⁎	0.514⁎⁎
VotB4HI	0.215⁎⁎	0.376⁎⁎	0.429⁎⁎	0.450⁎⁎	0.426⁎⁎	1	0.512⁎⁎
VotAftHI	0.284⁎⁎	0.485⁎⁎	0.530⁎⁎	0.469⁎⁎	0.514⁎⁎	0.512⁎⁎	1

* Correlation is significant at 0.05 level (2-tailed).

⁎⁎ Correlation is significant at 0.01 level (2-tailed).

There were significant correlations between all Events and State as shown in Table 4. This can be explained by the different vaccination registration rates in different states in Malaysia. The state of Selangor topped the list followed by Kuala Lumpur and Perak. The laggard states were Perlis, Malacca and Kedah. The various Events on confidence levels (ConVac, ConEcoVac, ConGovCov and ConGovEco) were found to be correlated with State, indicating the sentiments of the people differed from state to state. The people in different states showed different reactions to the questions of casting their votes in the general election before (VotB4HI) and after (VotAftHI) herd immunity, respectively. This may be explained by fear of Covid-19 cases spike alike after the general election in Sabah in late September 2020 [4]. However, all these correlations were significantly weak. These weak significant correlations were observed in Income with the Events.

**Table 4:** Correlations between Events and Socio-demographic characteristics.

Events	Ethnicity	Gender	State	Area	Gen	Edu	Inc
VacReg	-0.012	-0.027	-0.102⁎⁎	0.018	-0.018	0.131⁎⁎	0.024
ConVac	-0.043	0.001	-0.237⁎⁎	0.107⁎⁎	0.062*	0.029	0.162⁎⁎
ConEcoVac	0.009	-0.005	-0.250⁎⁎	0.060*	0.056	0.053	0.125⁎⁎
ConGovCov	0.035	-0.036	-0.241⁎⁎	0.017	0.088⁎⁎	0.014	0.153⁎⁎
ConGovEco	0.006	-0.007	-0.225⁎⁎	0.038	0.078⁎⁎	-0.006	0.152⁎⁎
VotB4HI	0.029	-0.04	-0.199⁎⁎	0.153⁎⁎	0.105⁎⁎	0.015	0.181⁎⁎
VotAftHI	0.043	0.001	-0.168⁎⁎	-0.017	0.043	0.131⁎⁎	0.130⁎⁎

⁎ Correlation is significant at 0.05 level (2-tailed).

⁎⁎ Correlation is significant at 0.01 level (2-tailed).

In conclusion, although the data collected by the poll is limited, they can be used by researchers for further analysis and the future generations to understand the discussed events during the Covid-19 pandemic in Malaysia.

## Ethics Statements

This manuscript has not been published elsewhere or it is under consideration for publication for other journals. Ethical approval was obtained from the Institutional Ethics Committee of UCSI University and granted the approval code of IEC-2021-FBM-079. Participants were informed of the objectives and requirements of the survey, were informed of their anonymity and that by agreeing to answer the online questionnaires, they confirmed their participation voluntarily and automatically provided an informed consent.

## CRediT Author Statement

**Pek Chuen Khee:** Conceptualization, Methodology, Software; **Choy Kah Yung:** Data curation, Writing – original draft preparation; **Toh Kian Kok:** Supervision and Validation; **Cham Tat Huei:** Writing – review & editing.

## Declaration of Competing Interest

The authors declare that they have no known competing financial interests or personal relationships that could have appeared to influence the work reported in this paper.

## Data Availability

Dataset of Vaccination and confidence in the Malaysian government during Covid-19 pandemic (Original data) (Mendeley Data).
